# Diaqua­(1,4,7,10,13-penta­oxacyclo­penta­deca­ne)iron(II) bis­(μ-*cis*-1,2-dicyano-1,2-ethyl­enedithiol­ato)bis­[(*cis*-1,2-dicyano-1,2-ethyl­enedithiol­ato)ferrate(III)] 1,4,7,10,13-penta­oxacyclo­penta­decane disolvate

**DOI:** 10.1107/S1600536808036805

**Published:** 2008-11-20

**Authors:** Toshiki Yamaguchi, Shigeyuki Masaoka, Ken Sakai

**Affiliations:** a Department of Chemistry, Faculty of Science, Kyushu University, Hakozaki 6-10-1, Higashi-ku, Fukuoka 812-8581, Japan

## Abstract

The title compound, [Fe(C_10_H_20_O_5_)(H_2_O)_2_][Fe_2_(C_4_N_2_S_2_)_4_]·2C_10_H_20_O_5_, consists of an [Fe^II^(15-crown-5)(H_2_O)_2_]^2+^ cation, sandwiched between and O—H⋯O hydrogen bonded by two additional 15-crown-5 ether mol­ecules and two independent [Fe^III^(mnt)_2_]^−^ anions, where 15-crown-5 ether denotes 1,4,7,10,13-penta­oxacyclo­penta­decane and mnt denotes *cis*-1,2-dicyano-1,2-ethyl­enedithiol­ate. Each independent [Fe^III^(mnt)_2_]^−^ unit forms a centrosymmetric dimer supported by two inter­monomer Fe^III^—S bonds [Fe—S = 2.4715 (9) and 2.4452 (9) Å]. In the crystal structure, the dimers form one-dimensional π–π stacks along the *a* axis, with an inter­planar separation of 3.38 (6) Å.

## Related literature

For general background, see: Adams (1990 ▶); Frey (2002 ▶); Georgakaki *et al.* (2003 ▶); Gloaguen *et al.* (2001 ▶); Liu *et al.* (2005 ▶); McCleverty *et al.* (1967 ▶); Na *et al.* (2006 ▶); Nicolet *et al.* (1999 ▶); Peters *et al.* (1998 ▶); Sakata (2000 ▶); Sellmann *et al.* (1991 ▶); Sun *et al.* (2005 ▶); Trasatti (1972 ▶); Yamaguchi *et al.* (2008 ▶). For related structures, see: Hamilton & Bernal (1967 ▶); Hao *et al.* (2005 ▶).
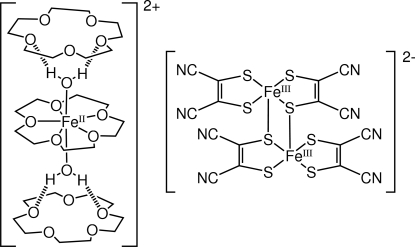


## Experimental

### 

#### Crystal data

[Fe(C_10_H_20_O_5_)(H_2_O)_2_][Fe_2_(C_4_N_2_S_2_)_4_]·2C_10_H_20_O_5_
*M* *_r_* = 1425.08
Monoclinic, 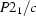

*a* = 13.376 (4) Å
*b* = 15.739 (4) Å
*c* = 30.069 (8) Å
β = 91.600 (4)°
*V* = 6328 (3) Å^3^
*Z* = 4
Mo *K*α radiation
μ = 1.01 mm^−1^
*T* = 100 (2) K
0.20 × 0.05 × 0.04 mm

#### Data collection

Bruker SMART APEX CCD area-detector diffractometer
Absorption correction: multi-scan (*SADABS*; Sheldrick, 1996 ▶) *T*_min_ = 0.742, *T*_max_ = 0.960
68765 measured reflections
13837 independent reflections
10591 reflections with *I* > 2σ(*I*)
*R*_int_ = 0.056

#### Refinement

*R*[*F* ^2^ > 2σ(*F* ^2^)] = 0.036
*wR*(*F* ^2^) = 0.073
*S* = 1.04
13837 reflections
755 parameters
H atoms treated by a mixture of independent and constrained refinement
Δρ_max_ = 0.55 e Å^−3^
Δρ_min_ = −0.34 e Å^−3^

### 

Data collection: *APEX2* (Bruker, 2007 ▶); cell refinement: *SAINT* (Bruker, 2007 ▶); data reduction: *SAINT*; program(s) used to solve structure: *SHELXS97* (Sheldrick, 2008 ▶); program(s) used to refine structure: *SHELXL97* (Sheldrick, 2008 ▶); molecular graphics: *KENX* (Sakai, 2004 ▶); software used to prepare material for publication: *SHELXL97*, *TEXSAN* (Molecular Structure Corporation, 2001 ▶), *KENX* and *ORTEPII* (Johnson, 1976 ▶).

## Supplementary Material

Crystal structure: contains datablocks global, I. DOI: 10.1107/S1600536808036805/lh2727sup1.cif

Structure factors: contains datablocks I. DOI: 10.1107/S1600536808036805/lh2727Isup2.hkl

Additional supplementary materials:  crystallographic information; 3D view; checkCIF report

## Figures and Tables

**Table 1:** Hydrogen-bond geometry (Å, °)

*D*—H⋯*A*	*D*—H	H⋯*A*	*D*⋯*A*	*D*—H⋯*A*
O6—H1⋯O8	0.78 (3)	1.96 (3)	2.726 (3)	171 (3)
O6—H2⋯O10	0.76 (3)	2.13 (3)	2.882 (2)	173 (3)
O7—H3⋯O13	0.76 (3)	2.04 (3)	2.779 (2)	164 (3)
O7—H4⋯O16	0.81 (3)	1.94 (3)	2.740 (2)	170 (3)

**Fig. 1.**
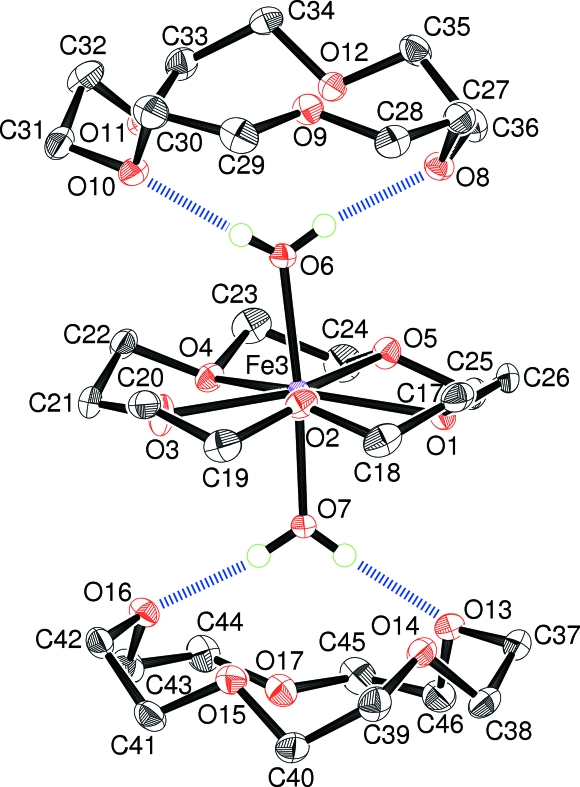
The structure of the [FeII(15-crown-5)3]2+ cation showing the atom-labeling scheme. Hydrogen atoms except for those of water molecules are omitted for clarity. Thermal ellipsoids are displayed at the 50% probability. Dashed lines indicate hydrogen bonds.

**Fig. 2.**
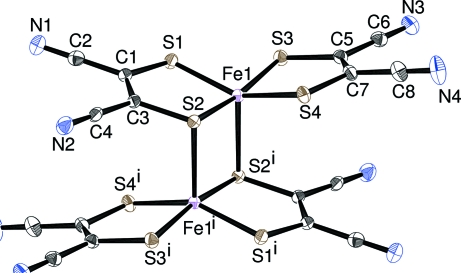
The crystal structure of one independent dimer of [FeIII(mnt)2]22-, showing the atom-labeling scheme [symmetry codes: (i) 1 - x, 1 - y, -z]. Thermal ellipsoids are displayed at the 50% probability.

**Fig. 3.**
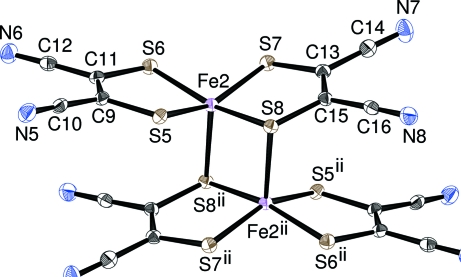
The structure of the second independent dimer of [FeIII(mnt)2]22-, showing the atom-labeling scheme [symmetry codes: (ii) -x, 1 - y, -z]. Thermal ellipsoids are displayed at the 50% probability.

**Fig. 4.**
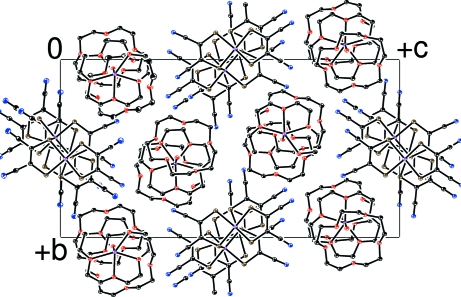
A view along the a axis, showing the manner in which the cations and anions separately stack along the a axis to give one-dimensional columns. Hydrogen atoms are omitted for clarity. Thermal ellipsoids are displayed at the 50% probability.

**Fig. 5.**
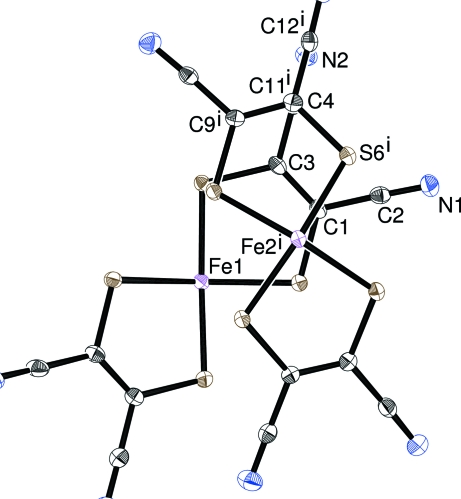
A view perpendicular to the plane defined by atoms C1—C4/N1—N2 which has a π-stack to the plane defined by atoms C9i, C11i, N12i and S6i [Symmetry code for (i) 1 - x, 1 - y, -z]. Thermal ellipsoids are displayed at the 50% probability.

## supplementary crystallographic information

### Comment

The Fe_2_S_2_ clusters (*i.e.*, H-clusters) in Fe-only hydrogenases (FeHases) are known to be highly active as catalysts towards hydrogen evolution reaction (HER) (Adams, 1990; Peters *et al.*, 1998; Nicolet *et al.*, 1999; Frey, 2002), in spite of the fact that metal iron itself exhibits much lower catalytic activity toward HER than platinum does (Trasatti, 1972; Sakata, 2000). A large variety of structural and functional models of FeHases have been developed and their H_2_-evolving activities have been evaluated so far (Gloaguen *et al.*, 2001; Georgakaki *et al.*, 2003; Liu *et al.*, 2005; Sun *et al.*, 2005). However, up to now, only two water-soluble models of FeHases have been ascertained to exhibit H_2_-evolving activity in aqueous media, even though their activities are still quite low (Na *et al.*, 2006). On the other hand, an iron-dithiolene complex, [Fe^II^(1,2-benzenedithiolato-S,*S*)_2_]^2-^, considered as a bio-inspired model, was found to generate a half equivalent of H_2_ in tetrahydrofurane in the presence of HCl (Sellmann *et al.*, 1991). In order to develop the more highly effective models of FeHases, our recent interests concentrate on such iron-dithiolene complexes, which are both air-stable and water-soluble. Compound (I) reported herein has been developed to improve the water-solubility of (NBu_4_)[Fe^III^(mnt)_2_] (Hamilton & Bernal, 1967). Although the sodium salt Na[Fe^III^(mnt)_2_] (McCleverty *et al.*, 1967) is soluble in water, the compound prepared by the literature method was found to involve a large amount of impurities. Thus, the improvement in the purity of the complex was another reason to develop a new water-soluble salt of this complex. The H_2_-evolving activity of (I) will be separately reported elsewhere (Yamaguchi *et al.*, unpublished results).

The asymmetric unit consists of a [Fe^II^(H_2_O)_2_(15-crown-5)_3_]^2+^ cation (Fig. 1) and two [Fe^III^(mnt)_2_]^-^ anions (Figs. 2 and 3). The oxidation states of these iron centers can be unambiguously judged from the overall charge of each complex together with the neutralization principle applied to any salt. The validity of these assignments can also be discussed in terms of the Fe—O and Fe—S distances (see below).

The Fe^II^ ion encapsulated within the central 15-crown-5 ether is ligated by five oxygen atoms of the ether and also by two oxygen atoms of axial aqua ligands (Fig. 1). The central [Fe^II^(H_2_O)_2_(15-crown-5)]^2+^ unit is sandwiched by two additional 15-crown-5 ether molecules, where each association is stabilized with two hydrogen bonds formed between the axial aqua ligand and two oxygen atoms of 15-crown-5 ether (see Table 1 and Fig. 1). The Fe^II^—O(15-crown-5) distances in (I) [2.1884 (17)–2.2367 (17) Å] are comparable to those reported for [Fe^II^(H_2_O)_2_(15-crown-5)](NO_3_)_2_ [2.187 (4)–2.246 (4) Å] (Hao *et al.*, 2005). Note that this is the second example showing the structure of [Fe^II^(H_2_O)_2_(15-crown-5)]^2+^. The Fe^II^—*O*(aqua) distances in (I) [2.0490 (17) and 2.0818 (17) Å] are similarly comparable to those reported for [Fe^II^(H_2_O)_2_(15-crown-5)](NO_3_)_2_ [2.063 (5) and 2.071 (5) Å] (Hao *et al.*, 2005).

The two independent mononuclear [Fe^III^(mnt)_2_]^-^ units respectively form a dimer with an inversion center located at the center of each dimer (see Figs. 2 and 3). The monomer-monomer association is supported by two Fe^III^—S bonds [Fe1—S2^i^ = 2.4715 (9) and Fe2—S8^ii^ = 2.4452 (9) Å; symmetry codes: (i) 1 - *x*, 1 - *y*, -*z*; (ii) -*x*, 1 - *y*, -*z*]. This structural feature well resembles those observed for (NBu_4_)[Fe^III^(mnt)_2_] [Fe—S(intermonomer) = 2.46 Å, where the estimated standard deviation is not given in the literature] (Hamilton & Bernal, 1967). Both Fe^III^ ions are considered to have a distorted square pyramidal coordination geometry. The Fe^III^ ion is ligated by four sulfur atoms with shorter Fe—S distances [2.2240 (7)–2.2447 (8) Å] and axially ligated by a sulfur atom from the adjacent monomer with a longer Fe—S distance [2.4452 (9) and 2.4715 (9) Å]. Atom Fe1 is shifted out of the least-squares plane defined with four atoms S1—S4 by 0.3634 (4) Å, even though the four-atom r.m.s. deviation given in the calculation was 0.177 Å. In the same manner, atom Fe2 is shifted out of the pseudo plane defined with S5—S8 by 0.3858 (4) Å, where the four-atom r.m.s. deviation was 0.104 Å.

The dihedral angle between the C1—C4/N1—N2 and C5—C8/N3—N4 planes is 21.40 (5)°, while that between the C9—C12/N5—N6 and C13—C16/N7—N8 planes is 20.03 (5)°. Shifts of sulfur atoms from the corresponding C_4_N_2_ plane are relatively large, where shifts of atoms S1—S8 from the individual plane are calculated to be 0.090 (3), 0.027 (3), 0.034 (3), 0.009 (3), 0.040 (3), 0.065 (3), 0.107 (3), and 0.003 (3) Å, respectively.

On the other hand, it is also important to compare the structural features of (I) with those of the H-clusters in FeHases. At the fully oxidized state, the Fe—Fe distance in the H-cluster from Clostridium pasteurianum was reported to be *ca* 2.62 Å (Peters *et al.*, 1998), which is much shorter than those observed for (I) [Fe1—Fe1^i^ = 3.2015 (9) Å, Fe2—Fe2^ii^ = 2.9939 (8) Å; symmetry codes: (i) 1 - *x*, 1 - *y*, -*z*; (ii) -*x*, 1 - *y*, -*z*]. Therefore, the metal-metal interactions in (I) is much weaker than those found in the H-cluster. The Fe—S—Fe angles in (I) [Fe1—S2—Fe1^i^ = 85.36 (2)°, Fe2—S8—Fe2^ii^ = 79.47 (2)°; symmetry codes: (i) 1 - *x*, 1 - *y*, -*z*; (ii) -*x*, 1 - *y*, -*z*] are much larger than the value of *ca* 68.4° observed for the H-cluster (Peters *et al.*, 1998), which also reflects that the metal-metal interaction in the H-cluster is stronger than those in (I). On the other hand, the average Fe—S distance in the H-cluster (*ca* 2.23 Å; Peters *et al.*, 1998) is comparable to the intramonomer Fe—S distances in (I) [2.2240 (7)–2.2447 (8) Å] but is much shorter than the intermonomer Fe—S distances in (I) [2.4452 (9)–2.4715 (9) Å].

Finally, the cations and anions separately form their individual one-dimensional stacks along the *a* axis (see Figure 4). The stack of cations merely arise from the van der Waals interactions, while that of anions is stabilized with a relatively strong π-π stacking interactions formed between two adjacent mnt moieties, where only one independent stacking geometry can be found in the crystal. As shown in Figure 5, a set of atoms C1—C4/N1—N2 and that of C9^i^, C11^i^, N12^i^, S6^i^ have a significant contribution to the π-π association at this geometry. The interplanar separation is calculated as 3.376 (55) Å based on the average shift of atoms C9^i^, C11^i^, N12^i^ and S6^i^ from the best plane defined by atoms C1—C4/N1—N2, and important short contacts at this geometry are C4—C11^i^ = 3.371 (3) and N2—C12^i^ = 3.324 (3) Å [Symmetry code for (i) 1 - *x*, 1 - *y*, -*z*].

### Experimental

Compound (I) was prepared as follows. Na[Fe^III^(mnt)_2_]^.^3H_2_O was prepared as previously described (McCleverty *et al.*, 1967). To a solution of Na[Fe^III^(mnt)_2_]^.^3H_2_O (0.108 g, 0.26 mmol) in ethanol (15 ml) was added 15-crown-5 ether (0.209 g, 0.95 mmol). The resulting dark-brown solution was stirred for 5 min and evaporated under reduced pressure until crystallization started. Standing of the solution at room temperature for 4 days afforded the black needles of (I), which were collected by filtration, washed with cold ethanol, and dried *in vacuo*. Yield: 0.072 g (39%). Since the starting material contains about 30% of Fe^II^ species (revealed by Mössbauer spectroscopy, Yamaguchi *et al.*, unpublished results), Fe^II^ ions are clathrated by 15-crown-5 ether molecules in the cations. Analysis calculated for C_46_H_64_Fe_3_N_8_O_17_S_8_: C, 38.77; H, 4.53; N, 7.86. Found: C, 38.64; H, 4.50; N, 7.96. IR (ν, cm^-1^): 3360 (w), 3265 (w), 2872 (w), 2216 (w), 2204 (*m*), 1657 (w), 1488 (*m*), 1472 (w), 1456 (w), 1353 (*m*), 1302 (w), 1290 (w), 1276 (w), 1249 (*m*), 1141 (*m*), 1118 (*s*), 1083 (*s*), 1039 (*s*), 961 (*s*), 937 (*s*), 850 (*m*), 835 (*m*), 608 (w), 546 (w), 505 (*s*), 432 (w), 420 (w), 411 (w).

### Refinement

H atoms except for those of water molecules were placed in idealized positions (methylene C—H = 0.99 Å), and included in the refinement in a riding-model approximation, with *U*_iso_(H) = 1.2Ueq (methylene C). H atoms of water molecules were refined isotropically. The hydrogen bonding geometries of these H atoms well support the validity of the positions determined by the least-squares calculations. In the final difference Fourier map, the highest peak was located 0.92 Å from atom Fe1. The deepest hole was located 0.72 Å from atom Fe1.

### Crystal data

**Table:**

[Fe(C_10_H_20_O_5_)(H_2_O)_2_][Fe_2_(C_4_N_2_S_2_)_4_]·2C_10_H_20_O_5_	*F*(000) = 2952
*M**_r_* = 1425.08	*D*_x_ = 1.496 Mg m^−^^3^
Monoclinic, *P*2_1_/*c*	Mo *K*α radiation, λ = 0.71073 Å
Hall symbol: -P 2ybc	Cell parameters from 9808 reflections
*a* = 13.376 (4) Å	θ = 2.4–27.5°
*b* = 15.739 (4) Å	µ = 1.01 mm^−^^1^
*c* = 30.069 (8) Å	*T* = 100 K
β = 91.600 (4)°	Needles, black
*V* = 6328 (3) Å^3^	0.20 × 0.05 × 0.04 mm
*Z* = 4	

### Data collection

**Table:**

Bruker SMART APEX CCD area-detector diffractometer	13837 independent reflections
Radiation source: rotating anode with a mirror focusing unit	10591 reflections with *I* > 2σ(*I*)
graphite	*R*_int_ = 0.056
φ and ω scans	θ_max_ = 27.1°, θ_min_ = 2.1°
Absorption correction: multi-scan (*SADABS*; Sheldrick, 1996)	*h* = −17→17
*T*_min_ = 0.742, *T*_max_ = 0.960	*k* = −20→20
68765 measured reflections	*l* = −38→38

### Refinement

**Table:**

Refinement on *F*^2^	Primary atom site location: structure-invariant direct methods
Least-squares matrix: full	Secondary atom site location: difference Fourier map
*R*[*F*^2^ > 2σ(*F*^2^)] = 0.036	Hydrogen site location: inferred from neighbouring sites
*wR*(*F*^2^) = 0.073	H atoms treated by a mixture of independent and constrained refinement
*S* = 1.04	*w* = 1/[σ^2^(*F*_o_^2^) + (0.0267*P*)^2^ + 3.2078*P*] where *P* = (*F*_o_^2^ + 2*F*_c_^2^)/3
13837 reflections	(Δ/σ)_max_ = 0.001
755 parameters	Δρ_max_ = 0.55 e Å^−^^3^
0 restraints	Δρ_min_ = −0.34 e Å^−^^3^

### Special details

**Table:**

Experimental. The first 50 frames were rescanned at the end of data collection to evaluate any possible decay phenomenon. Since it was judged to be negligible, no decay correction was applied to the data.
Geometry. All e.s.d.'s (except the e.s.d. in the dihedral angle between two l.s. planes) are estimated using the full covariance matrix. The cell e.s.d.'s are taken into account individually in the estimation of e.s.d.'s in distances, angles and torsion angles; correlations between e.s.d.'s in cell parameters are only used when they are defined by crystal symmetry. An approximate (isotropic) treatment of cell e.s.d.'s is used for estimating e.s.d.'s involving l.s. planes.Least-squares planes (*x*,*y*,*z* in crystal coordinates) and deviations from them (* indicates atom used to define plane)12.5966 (0.0045) *x* + 3.9174 (0.0133) *y* - 7.5943 (0.0193) *z* = 9.5633 (0.0036)* -0.0136 (0.0015) C1 * 0.0089 (0.0018) C2 * 0.0060 (0.0015) C3 * 0.0086 (0.0018) C4 * -0.0017 (0.0012) N1 * -0.0081 (0.0012) N2 3.4170 (0.0029) C9_$1 3.3793 (0.0026) C11_$1 3.2979 (0.0031) C12_$1 3.4112 (0.0014) S6_$1Rms deviation of fitted atoms = 0.008613.1161 (0.0038) *x* + 0.5753 (0.0029) *y* - 6.6201 (0.0054) *z* = 8.3501 (0.0028)Angle to previous plane (with approximate e.s.d.) = 13.04 (0.05)* 0.1774 (0.0003) S1 * -0.1759 (0.0003) S2 * -0.1778 (0.0003) S3 * 0.1763 (0.0003) S4 - 0.3634 (0.0004) Fe1Rms deviation of fitted atoms = 0.176812.9276 (0.0037) *x* + 2.2639 (0.0028) *y* - 7.2069 (0.0056) *z* = 2.5969 (0.0017)Angle to previous plane (with approximate e.s.d.) = 6.31 (0.02)* 0.1070 (0.0003) S5 * -0.1009 (0.0003) S6 * 0.1003 (0.0003) S7 * -0.1064 (0.0003) S8 - 0.3858 (0.0004) Fe2Rms deviation of fitted atoms = 0.103712.5966 (0.0045) *x* + 3.9175 (0.0133) *y* - 7.5943 (0.0193) *z* = 9.5633 (0.0036)Angle to previous plane (with approximate e.s.d.) = 6.24 (0.04)* -0.0136 (0.0015) C1 * 0.0089 (0.0018) C2 * 0.0060 (0.0015) C3 * 0.0086 (0.0018) C4 * -0.0017 (0.0012) N1 * -0.0081 (0.0012) N2 - 0.0896 (0.0028) S1 - 0.0274 (0.0028) S2Rms deviation of fitted atoms = 0.008613.2007 (0.0042) *x* - 1.6498 (0.0149) *y* - 4.5212 (0.0193) *z* = 7.1258 (0.0133)Angle to previous plane (with approximate e.s.d.) = 21.40 (0.05)* 0.0089 (0.0016) C5 * 0.0023 (0.0018) C6 * -0.0072 (0.0016) C7 * -0.0063 (0.0020) C8 * -0.0046 (0.0012) N3 * 0.0069 (0.0013) N4 0.0344 (0.0030) S3 - 0.0088 (0.0028) S4Rms deviation of fitted atoms = 0.006412.4166 (0.0053) *x* + 3.7221 (0.0089) *y* - 9.4088 (0.0267) *z* = 3.2121 (0.0070)Angle to previous plane (with approximate e.s.d.) = 22.10 (0.05)* -0.0149 (0.0016) C9 * 0.0022 (0.0018) C10 * 0.0105 (0.0016) C11 * 0.0072 (0.0018) C12 * 0.0036 (0.0012) N5 * -0.0086 (0.0012) N6 - 0.0398 (0.0028) S5 0.0647 (0.0028) S6Rms deviation of fitted atoms = 0.008913.3465 (0.0038) *x* + 1.0421 (0.0093) *y* - 0.5940 (0.0281) *z* = 1.7628 (0.0060)Angle to previous plane (with approximate e.s.d.) = 20.03 (0.05)* -0.0211 (0.0015) C13 * -0.0038 (0.0019) C14 * 0.0151 (0.0015) C15 * 0.0182 (0.0018) C16 * 0.0098 (0.0012) N7 * -0.0181 (0.0012) N8 - 0.1066 (0.0028) S7 0.0032 (0.0028) S8Rms deviation of fitted atoms = 0.0155
Refinement. Refinement of *F*^2^ against ALL reflections. The weighted *R*-factor *wR* and goodness of fit *S* are based on *F*^2^, conventional *R*-factors *R* are based on *F*, with *F* set to zero for negative *F*^2^. The threshold expression of *F*^2^ > σ(*F*^2^) is used only for calculating *R*-factors(gt) *etc*. and is not relevant to the choice of reflections for refinement. *R*-factors based on *F*^2^ are statistically about twice as large as those based on *F*, and *R*- factors based on ALL data will be even larger.

### Fractional atomic coordinates and isotropic or equivalent isotropic displacement parameters (Å^2^)

**Table:**

	*x*	*y*	*z*	*U*_iso_*/*U*_eq_	
Fe1	0.59954 (2)	0.527018 (19)	0.027212 (10)	0.01380 (7)	
Fe2	0.08164 (2)	0.559112 (19)	0.015272 (11)	0.01436 (7)	
Fe3	0.22188 (2)	0.081610 (19)	0.160542 (10)	0.01279 (7)	
S1	0.65583 (4)	0.39842 (4)	0.045857 (19)	0.01683 (12)	
S2	0.57986 (4)	0.48479 (3)	−0.043795 (18)	0.01451 (11)	
S3	0.64623 (4)	0.56941 (4)	0.095356 (19)	0.01793 (12)	
S4	0.62207 (4)	0.65871 (4)	0.001767 (19)	0.01773 (12)	
S5	0.16136 (4)	0.47243 (3)	0.062681 (19)	0.01600 (12)	
S6	0.11068 (4)	0.66775 (3)	0.061957 (19)	0.01783 (12)	
S7	0.07146 (4)	0.65027 (3)	−0.041804 (19)	0.01742 (12)	
S8	0.09565 (4)	0.45117 (3)	−0.032270 (18)	0.01515 (11)	
O1	0.19270 (11)	0.12133 (10)	0.08849 (5)	0.0193 (3)	
O2	0.27147 (11)	−0.02428 (10)	0.11811 (5)	0.0197 (3)	
O3	0.23720 (13)	−0.03008 (10)	0.20354 (5)	0.0270 (4)	
O4	0.19983 (11)	0.12270 (10)	0.23080 (5)	0.0196 (3)	
O5	0.20035 (11)	0.22228 (10)	0.15824 (5)	0.0206 (4)	
O6	0.37295 (12)	0.10924 (12)	0.17086 (6)	0.0191 (4)	
O7	0.07301 (12)	0.05129 (12)	0.15570 (6)	0.0207 (4)	
O8	0.44831 (11)	0.21480 (10)	0.10820 (5)	0.0200 (3)	
O9	0.57976 (11)	0.07865 (10)	0.13190 (5)	0.0218 (4)	
O10	0.51901 (11)	0.00073 (10)	0.21446 (5)	0.0218 (4)	
O11	0.49149 (11)	0.15375 (10)	0.26444 (6)	0.0244 (4)	
O12	0.48824 (12)	0.28340 (10)	0.19532 (5)	0.0240 (4)	
O13	−0.07486 (11)	0.12746 (10)	0.10257 (5)	0.0215 (4)	
O14	−0.01589 (12)	−0.03072 (10)	0.05980 (5)	0.0236 (4)	
O15	−0.01408 (12)	−0.14420 (10)	0.13565 (5)	0.0248 (4)	
O16	0.00158 (11)	−0.05479 (10)	0.21969 (5)	0.0208 (4)	
O17	−0.14297 (12)	0.06099 (10)	0.18609 (6)	0.0240 (4)	
N1	0.70216 (15)	0.18374 (13)	0.00039 (7)	0.0255 (5)	
N2	0.59855 (15)	0.29093 (13)	−0.11533 (7)	0.0262 (5)	
N3	0.69081 (15)	0.76836 (13)	0.16152 (7)	0.0254 (5)	
N4	0.66447 (19)	0.88016 (14)	0.04131 (7)	0.0365 (6)	
N5	0.25737 (15)	0.45610 (13)	0.17831 (7)	0.0275 (5)	
N6	0.18424 (16)	0.70649 (13)	0.18215 (7)	0.0273 (5)	
N7	0.07441 (17)	0.65303 (14)	−0.16666 (7)	0.0315 (5)	
N8	0.09250 (16)	0.40171 (13)	−0.15397 (7)	0.0285 (5)	
C1	0.64947 (15)	0.34198 (14)	−0.00380 (8)	0.0164 (5)	
C2	0.67963 (16)	0.25395 (15)	−0.00215 (7)	0.0188 (5)	
C3	0.61651 (15)	0.37748 (14)	−0.04274 (7)	0.0154 (5)	
C4	0.60718 (16)	0.32967 (14)	−0.08322 (8)	0.0175 (5)	
C5	0.65634 (16)	0.67935 (14)	0.09037 (7)	0.0171 (5)	
C6	0.67553 (16)	0.72830 (15)	0.13001 (8)	0.0197 (5)	
C7	0.64611 (16)	0.71784 (14)	0.05002 (8)	0.0178 (5)	
C8	0.65594 (18)	0.80839 (16)	0.04549 (8)	0.0237 (5)	
C9	0.18064 (15)	0.53441 (14)	0.10999 (7)	0.0164 (5)	
C10	0.22371 (16)	0.49240 (14)	0.14840 (8)	0.0186 (5)	
C11	0.15743 (16)	0.61876 (14)	0.11004 (7)	0.0177 (5)	
C12	0.17233 (17)	0.66872 (14)	0.14981 (8)	0.0193 (5)	
C13	0.08089 (15)	0.58501 (14)	−0.08816 (7)	0.0165 (5)	
C14	0.07722 (17)	0.62397 (15)	−0.13157 (8)	0.0208 (5)	
C15	0.09045 (15)	0.49926 (14)	−0.08495 (7)	0.0159 (5)	
C16	0.09320 (16)	0.44502 (15)	−0.12327 (8)	0.0184 (5)	
C17	0.24863 (18)	0.07096 (16)	0.05811 (8)	0.0253 (6)	
H5	0.3201	0.0875	0.0594	0.030*	
H6	0.2224	0.0788	0.0273	0.030*	
C18	0.23663 (18)	−0.01932 (16)	0.07239 (8)	0.0265 (6)	
H7	0.1655	−0.0365	0.0697	0.032*	
H8	0.2765	−0.0574	0.0536	0.032*	
C19	0.25546 (18)	−0.10670 (15)	0.13776 (9)	0.0265 (6)	
H9	0.2941	−0.1506	0.1221	0.032*	
H10	0.1837	−0.1220	0.1357	0.032*	
C20	0.29009 (17)	−0.10070 (15)	0.18559 (8)	0.0243 (5)	
H11	0.2742	−0.1536	0.2018	0.029*	
H12	0.3632	−0.0910	0.1878	0.029*	
C21	0.23440 (18)	−0.01912 (16)	0.25106 (8)	0.0260 (6)	
H13	0.2840	−0.0569	0.2660	0.031*	
H14	0.1672	−0.0338	0.2617	0.031*	
C22	0.25811 (17)	0.07187 (16)	0.26164 (8)	0.0233 (5)	
H15	0.2404	0.0853	0.2926	0.028*	
H16	0.3303	0.0830	0.2583	0.028*	
C23	0.21768 (19)	0.21235 (15)	0.23597 (9)	0.0274 (6)	
H17	0.2900	0.2250	0.2342	0.033*	
H18	0.1941	0.2323	0.2651	0.033*	
C24	0.16045 (19)	0.25483 (15)	0.19884 (8)	0.0275 (6)	
H19	0.0883	0.2415	0.2004	0.033*	
H20	0.1691	0.3172	0.2005	0.033*	
C25	0.14642 (18)	0.25152 (15)	0.11908 (8)	0.0251 (6)	
H21	0.1497	0.3142	0.1169	0.030*	
H22	0.0754	0.2342	0.1199	0.030*	
C26	0.19651 (18)	0.21083 (15)	0.08030 (8)	0.0245 (6)	
H23	0.1606	0.2251	0.0521	0.029*	
H24	0.2667	0.2303	0.0786	0.029*	
C27	0.52881 (17)	0.18696 (16)	0.08105 (8)	0.0242 (5)	
H25	0.5908	0.2182	0.0895	0.029*	
H26	0.5124	0.1989	0.0494	0.029*	
C28	0.54479 (18)	0.09321 (16)	0.08753 (8)	0.0256 (5)	
H27	0.4812	0.0624	0.0819	0.031*	
H28	0.5945	0.0721	0.0664	0.031*	
C29	0.58859 (19)	−0.00964 (15)	0.14211 (9)	0.0290 (6)	
H29	0.6440	−0.0349	0.1254	0.035*	
H30	0.5260	−0.0395	0.1333	0.035*	
C30	0.60860 (18)	−0.01933 (16)	0.19102 (9)	0.0280 (6)	
H31	0.6295	−0.0784	0.1978	0.034*	
H32	0.6634	0.0193	0.2008	0.034*	
C31	0.53724 (18)	0.00910 (16)	0.26145 (8)	0.0264 (6)	
H33	0.5888	−0.0327	0.2711	0.032*	
H34	0.4750	−0.0044	0.2771	0.032*	
C32	0.57162 (18)	0.09675 (16)	0.27461 (9)	0.0301 (6)	
H35	0.5890	0.0986	0.3068	0.036*	
H36	0.6315	0.1128	0.2579	0.036*	
C33	0.51659 (18)	0.24121 (15)	0.27080 (8)	0.0252 (5)	
H37	0.5648	0.2462	0.2962	0.030*	
H38	0.4555	0.2730	0.2784	0.030*	
C34	0.56127 (18)	0.28076 (16)	0.23036 (8)	0.0261 (6)	
H39	0.5845	0.3390	0.2375	0.031*	
H40	0.6196	0.2470	0.2211	0.031*	
C35	0.52417 (19)	0.32257 (16)	0.15643 (8)	0.0260 (6)	
H41	0.5910	0.2999	0.1496	0.031*	
H42	0.5300	0.3847	0.1610	0.031*	
C36	0.45148 (18)	0.30401 (15)	0.11875 (8)	0.0232 (5)	
H43	0.3839	0.3231	0.1269	0.028*	
H44	0.4710	0.3364	0.0921	0.028*	
C37	−0.07617 (18)	0.11119 (15)	0.05578 (8)	0.0244 (5)	
H45	−0.0103	0.1266	0.0439	0.029*	
H46	−0.1272	0.1479	0.0410	0.029*	
C38	−0.09861 (18)	0.01978 (16)	0.04446 (9)	0.0275 (6)	
H47	−0.1604	0.0014	0.0590	0.033*	
H48	−0.1086	0.0132	0.0119	0.033*	
C39	−0.03454 (19)	−0.12009 (15)	0.05793 (8)	0.0267 (6)	
H49	0.0296	−0.1501	0.0538	0.032*	
H50	−0.0786	−0.1324	0.0317	0.032*	
C40	−0.08261 (18)	−0.15415 (16)	0.09902 (8)	0.0265 (6)	
H51	−0.1451	−0.1226	0.1046	0.032*	
H52	−0.0994	−0.2150	0.0949	0.032*	
C41	−0.05309 (18)	−0.17727 (15)	0.17582 (8)	0.0249 (5)	
H53	−0.0521	−0.2402	0.1752	0.030*	
H54	−0.1231	−0.1584	0.1791	0.030*	
C42	0.01075 (17)	−0.14521 (14)	0.21404 (8)	0.0225 (5)	
H55	−0.0091	−0.1741	0.2417	0.027*	
H56	0.0815	−0.1595	0.2089	0.027*	
C43	−0.08396 (18)	−0.02924 (16)	0.24395 (8)	0.0273 (6)	
H57	−0.0690	−0.0331	0.2763	0.033*	
H58	−0.1411	−0.0672	0.2367	0.033*	
C44	−0.10995 (19)	0.06062 (16)	0.23150 (8)	0.0272 (6)	
H59	−0.1635	0.0821	0.2506	0.033*	
H60	−0.0506	0.0977	0.2356	0.033*	
C45	−0.16276 (19)	0.14463 (16)	0.16992 (9)	0.0300 (6)	
H61	−0.1076	0.1833	0.1792	0.036*	
H62	−0.2256	0.1663	0.1823	0.036*	
C46	−0.17198 (17)	0.14146 (16)	0.12004 (9)	0.0279 (6)	
H63	−0.2177	0.0949	0.1107	0.033*	
H64	−0.1999	0.1957	0.1085	0.033*	
H4	0.049 (2)	0.0167 (18)	0.1723 (9)	0.033 (8)*	
H3	0.035 (2)	0.0648 (18)	0.1378 (10)	0.035 (9)*	
H2	0.409 (2)	0.0815 (19)	0.1842 (10)	0.037 (9)*	
H1	0.399 (2)	0.1409 (19)	0.1551 (10)	0.041 (10)*	

### Atomic displacement parameters (Å^2^)

**Table:**

	*U*^11^	*U*^22^	*U*^33^	*U*^12^	*U*^13^	*U*^23^
Fe1	0.01341 (15)	0.01369 (16)	0.01430 (17)	0.00033 (12)	0.00008 (12)	−0.00119 (13)
Fe2	0.01444 (15)	0.01257 (16)	0.01611 (17)	0.00023 (12)	0.00095 (12)	−0.00028 (13)
Fe3	0.01362 (15)	0.01173 (16)	0.01300 (17)	0.00013 (12)	−0.00025 (12)	−0.00072 (12)
S1	0.0191 (3)	0.0155 (3)	0.0158 (3)	0.0029 (2)	−0.0008 (2)	−0.0010 (2)
S2	0.0140 (2)	0.0144 (3)	0.0152 (3)	0.0007 (2)	0.0008 (2)	−0.0009 (2)
S3	0.0214 (3)	0.0159 (3)	0.0163 (3)	0.0000 (2)	−0.0032 (2)	−0.0003 (2)
S4	0.0228 (3)	0.0159 (3)	0.0145 (3)	−0.0029 (2)	0.0004 (2)	−0.0008 (2)
S5	0.0164 (3)	0.0136 (3)	0.0180 (3)	0.0012 (2)	−0.0013 (2)	−0.0015 (2)
S6	0.0212 (3)	0.0131 (3)	0.0192 (3)	−0.0005 (2)	0.0000 (2)	−0.0008 (2)
S7	0.0203 (3)	0.0134 (3)	0.0186 (3)	0.0008 (2)	0.0008 (2)	−0.0001 (2)
S8	0.0156 (3)	0.0132 (3)	0.0167 (3)	0.0005 (2)	0.0017 (2)	0.0000 (2)
O1	0.0208 (8)	0.0205 (9)	0.0166 (9)	−0.0025 (6)	0.0017 (6)	0.0023 (7)
O2	0.0197 (8)	0.0178 (8)	0.0215 (9)	0.0014 (6)	−0.0026 (7)	−0.0053 (7)
O3	0.0410 (10)	0.0182 (9)	0.0214 (10)	0.0067 (7)	−0.0031 (8)	0.0036 (7)
O4	0.0199 (8)	0.0226 (9)	0.0162 (9)	0.0002 (7)	−0.0019 (6)	−0.0023 (7)
O5	0.0245 (8)	0.0148 (8)	0.0223 (9)	0.0025 (6)	−0.0028 (7)	−0.0008 (7)
O6	0.0145 (8)	0.0197 (9)	0.0231 (10)	−0.0012 (7)	−0.0006 (7)	0.0048 (8)
O7	0.0144 (8)	0.0258 (10)	0.0218 (10)	−0.0038 (7)	−0.0025 (7)	0.0086 (8)
O8	0.0184 (8)	0.0218 (9)	0.0198 (9)	−0.0009 (6)	0.0026 (7)	−0.0002 (7)
O9	0.0225 (8)	0.0185 (9)	0.0243 (9)	0.0013 (7)	−0.0003 (7)	−0.0012 (7)
O10	0.0181 (8)	0.0227 (9)	0.0244 (9)	0.0002 (7)	−0.0027 (7)	0.0017 (7)
O11	0.0221 (8)	0.0206 (9)	0.0302 (10)	−0.0007 (7)	−0.0068 (7)	0.0013 (7)
O12	0.0249 (9)	0.0296 (10)	0.0174 (9)	−0.0071 (7)	−0.0028 (7)	0.0021 (7)
O13	0.0162 (8)	0.0246 (9)	0.0236 (9)	−0.0002 (7)	−0.0012 (7)	0.0020 (7)
O14	0.0240 (9)	0.0213 (9)	0.0249 (10)	−0.0017 (7)	−0.0060 (7)	0.0018 (7)
O15	0.0244 (9)	0.0274 (10)	0.0225 (10)	−0.0070 (7)	−0.0017 (7)	0.0017 (7)
O16	0.0192 (8)	0.0203 (9)	0.0232 (9)	−0.0007 (7)	0.0045 (7)	0.0017 (7)
O17	0.0240 (9)	0.0218 (9)	0.0264 (10)	0.0007 (7)	0.0042 (7)	0.0007 (7)
N1	0.0302 (11)	0.0200 (11)	0.0264 (12)	0.0057 (9)	0.0026 (9)	−0.0006 (9)
N2	0.0280 (11)	0.0267 (12)	0.0238 (12)	0.0058 (9)	−0.0014 (9)	−0.0058 (10)
N3	0.0285 (11)	0.0254 (12)	0.0222 (12)	−0.0046 (9)	−0.0010 (9)	−0.0026 (9)
N4	0.0644 (17)	0.0216 (13)	0.0237 (13)	−0.0103 (11)	0.0075 (11)	−0.0019 (10)
N5	0.0281 (11)	0.0271 (12)	0.0270 (12)	−0.0027 (9)	−0.0071 (9)	0.0010 (10)
N6	0.0383 (12)	0.0194 (11)	0.0240 (12)	−0.0025 (9)	−0.0009 (10)	−0.0031 (9)
N7	0.0469 (14)	0.0255 (12)	0.0220 (13)	0.0032 (10)	−0.0016 (10)	0.0027 (10)
N8	0.0339 (12)	0.0273 (12)	0.0245 (12)	−0.0063 (9)	0.0067 (9)	−0.0048 (10)
C1	0.0131 (10)	0.0155 (11)	0.0207 (13)	0.0008 (9)	0.0011 (9)	−0.0022 (9)
C2	0.0168 (11)	0.0238 (13)	0.0159 (12)	0.0007 (9)	0.0022 (9)	−0.0022 (10)
C3	0.0119 (10)	0.0147 (11)	0.0199 (12)	0.0001 (8)	0.0044 (9)	−0.0035 (9)
C4	0.0158 (11)	0.0155 (12)	0.0212 (13)	0.0037 (9)	0.0019 (9)	0.0008 (10)
C5	0.0148 (11)	0.0177 (12)	0.0188 (12)	−0.0014 (9)	0.0012 (9)	−0.0023 (9)
C6	0.0180 (11)	0.0210 (12)	0.0201 (13)	−0.0015 (9)	0.0003 (9)	0.0023 (10)
C7	0.0170 (11)	0.0180 (12)	0.0184 (12)	−0.0032 (9)	0.0019 (9)	−0.0038 (10)
C8	0.0313 (13)	0.0243 (14)	0.0158 (13)	−0.0064 (10)	0.0043 (10)	−0.0024 (10)
C9	0.0137 (10)	0.0187 (12)	0.0167 (12)	−0.0020 (9)	0.0004 (9)	−0.0016 (9)
C10	0.0181 (11)	0.0168 (12)	0.0210 (13)	−0.0032 (9)	−0.0005 (10)	−0.0041 (10)
C11	0.0153 (11)	0.0187 (12)	0.0190 (12)	−0.0040 (9)	0.0014 (9)	−0.0009 (10)
C12	0.0213 (12)	0.0143 (12)	0.0224 (14)	−0.0015 (9)	0.0013 (10)	0.0029 (10)
C13	0.0126 (10)	0.0189 (12)	0.0180 (12)	0.0003 (9)	0.0009 (9)	0.0009 (9)
C14	0.0219 (12)	0.0177 (12)	0.0228 (14)	0.0024 (9)	0.0011 (10)	−0.0034 (10)
C15	0.0136 (10)	0.0185 (12)	0.0157 (12)	−0.0002 (9)	0.0015 (9)	0.0005 (9)
C16	0.0163 (11)	0.0204 (12)	0.0187 (13)	−0.0008 (9)	0.0030 (9)	0.0040 (10)
C17	0.0206 (12)	0.0409 (16)	0.0145 (13)	−0.0036 (11)	−0.0001 (10)	−0.0028 (11)
C18	0.0263 (13)	0.0321 (15)	0.0209 (14)	0.0024 (11)	−0.0012 (10)	−0.0128 (11)
C19	0.0261 (13)	0.0138 (12)	0.0395 (16)	0.0032 (10)	−0.0035 (11)	−0.0047 (11)
C20	0.0198 (12)	0.0149 (12)	0.0378 (16)	0.0006 (9)	−0.0051 (10)	0.0051 (11)
C21	0.0259 (13)	0.0328 (15)	0.0189 (13)	−0.0038 (11)	−0.0051 (10)	0.0103 (11)
C22	0.0194 (12)	0.0345 (15)	0.0158 (13)	0.0006 (10)	−0.0024 (9)	0.0034 (11)
C23	0.0318 (14)	0.0231 (13)	0.0273 (15)	0.0018 (11)	−0.0010 (11)	−0.0126 (11)
C24	0.0333 (14)	0.0181 (13)	0.0312 (15)	0.0049 (10)	0.0015 (11)	−0.0086 (11)
C25	0.0268 (13)	0.0158 (12)	0.0321 (15)	−0.0002 (10)	−0.0084 (11)	0.0076 (11)
C26	0.0267 (13)	0.0238 (13)	0.0225 (14)	−0.0069 (10)	−0.0068 (10)	0.0111 (11)
C27	0.0211 (12)	0.0324 (14)	0.0194 (13)	0.0003 (10)	0.0035 (10)	−0.0004 (11)
C28	0.0234 (12)	0.0305 (14)	0.0231 (14)	0.0020 (10)	0.0020 (10)	−0.0059 (11)
C29	0.0285 (13)	0.0206 (13)	0.0383 (16)	0.0032 (10)	0.0071 (12)	−0.0037 (11)
C30	0.0225 (12)	0.0206 (13)	0.0411 (17)	0.0049 (10)	0.0029 (11)	0.0044 (12)
C31	0.0260 (13)	0.0272 (14)	0.0256 (15)	0.0016 (10)	−0.0069 (11)	0.0041 (11)
C32	0.0251 (13)	0.0325 (15)	0.0320 (16)	0.0021 (11)	−0.0110 (11)	−0.0024 (12)
C33	0.0298 (13)	0.0253 (14)	0.0202 (13)	−0.0028 (10)	−0.0036 (10)	−0.0038 (11)
C34	0.0246 (13)	0.0291 (14)	0.0243 (14)	−0.0073 (10)	−0.0064 (10)	−0.0012 (11)
C35	0.0322 (14)	0.0237 (13)	0.0220 (14)	−0.0107 (11)	0.0006 (11)	−0.0002 (11)
C36	0.0287 (13)	0.0189 (12)	0.0219 (13)	−0.0007 (10)	0.0004 (10)	0.0034 (10)
C37	0.0232 (12)	0.0255 (13)	0.0241 (14)	−0.0008 (10)	−0.0048 (10)	0.0052 (11)
C38	0.0249 (13)	0.0317 (15)	0.0253 (14)	−0.0018 (11)	−0.0090 (11)	0.0006 (11)
C39	0.0329 (14)	0.0252 (14)	0.0217 (14)	−0.0023 (11)	−0.0038 (11)	−0.0045 (11)
C40	0.0289 (13)	0.0231 (13)	0.0273 (15)	−0.0067 (10)	−0.0062 (11)	−0.0016 (11)
C41	0.0281 (13)	0.0194 (13)	0.0273 (14)	−0.0072 (10)	0.0024 (11)	0.0021 (10)
C42	0.0232 (12)	0.0201 (13)	0.0243 (14)	−0.0007 (10)	0.0021 (10)	0.0056 (10)
C43	0.0250 (13)	0.0327 (15)	0.0246 (14)	0.0003 (11)	0.0083 (10)	0.0037 (11)
C44	0.0297 (14)	0.0296 (14)	0.0228 (14)	0.0028 (11)	0.0082 (11)	−0.0017 (11)
C45	0.0285 (14)	0.0230 (14)	0.0390 (17)	0.0059 (11)	0.0090 (12)	0.0027 (12)
C46	0.0187 (12)	0.0265 (14)	0.0386 (16)	0.0044 (10)	0.0023 (11)	0.0075 (12)

### Geometric parameters (Å, °)

**Table:**

Fe1—S1	2.2259 (8)	C13—C15	1.359 (3)
Fe1—S3	2.2276 (8)	C13—C14	1.442 (3)
Fe1—S4	2.2328 (8)	C15—C16	1.435 (3)
Fe1—S2	2.2447 (8)	C17—C18	1.494 (4)
Fe1—S2^i^	2.4715 (9)	C19—C20	1.502 (3)
Fe2—S5	2.2240 (7)	C21—C22	1.499 (3)
Fe2—S8	2.2316 (8)	C23—C24	1.494 (3)
Fe2—S7	2.2382 (8)	C25—C26	1.504 (3)
Fe2—S6	2.2394 (8)	C27—C28	1.503 (3)
Fe2—S8^ii^	2.4452 (9)	C29—C30	1.495 (4)
Fe3—O7	2.0490 (17)	C31—C32	1.504 (3)
Fe3—O6	2.0818 (17)	C33—C34	1.505 (3)
Fe3—O3	2.1884 (17)	C35—C36	1.501 (3)
Fe3—O2	2.2120 (16)	C37—C38	1.507 (3)
Fe3—O5	2.2335 (16)	C39—C40	1.507 (3)
Fe3—O4	2.2367 (17)	C41—C42	1.500 (3)
Fe3—O1	2.2782 (16)	C43—C44	1.501 (3)
S1—C1	1.737 (2)	C45—C46	1.502 (4)
S2—C3	1.759 (2)	C17—H5	0.9900
S2—Fe1^i^	2.4715 (9)	C17—H6	0.9900
S3—C5	1.742 (2)	C18—H7	0.9900
S4—C7	1.746 (2)	C18—H8	0.9900
S5—C9	1.738 (2)	C19—H9	0.9900
S6—C11	1.739 (2)	C19—H10	0.9900
S7—C13	1.739 (2)	C20—H11	0.9900
S8—C15	1.755 (2)	C20—H12	0.9900
S8—Fe2^ii^	2.4452 (9)	C21—H13	0.9900
O1—C26	1.431 (3)	C21—H14	0.9900
O1—C17	1.435 (3)	C22—H15	0.9900
O2—C18	1.441 (3)	C22—H16	0.9900
O2—C19	1.444 (3)	C23—H17	0.9900
O3—C20	1.431 (3)	C23—H18	0.9900
O3—C21	1.441 (3)	C24—H19	0.9900
O4—C22	1.438 (3)	C24—H20	0.9900
O4—C23	1.439 (3)	C25—H21	0.9900
O5—C25	1.439 (3)	C25—H22	0.9900
O5—C24	1.440 (3)	C26—H23	0.9900
O6—H2	0.76 (3)	C26—H24	0.9900
O6—H1	0.78 (3)	C27—H25	0.9900
O7—H4	0.81 (3)	C27—H26	0.9900
O7—H3	0.76 (3)	C28—H27	0.9900
O8—C27	1.438 (3)	C28—H28	0.9900
O8—C36	1.440 (3)	C29—H29	0.9900
O9—C28	1.420 (3)	C29—H30	0.9900
O9—C29	1.427 (3)	C30—H31	0.9900
O10—C31	1.433 (3)	C30—H32	0.9900
O10—C30	1.442 (3)	C31—H33	0.9900
O11—C32	1.424 (3)	C31—H34	0.9900
O11—C33	1.429 (3)	C32—H35	0.9900
O12—C34	1.417 (3)	C32—H36	0.9900
O12—C35	1.418 (3)	C33—H37	0.9900
O13—C37	1.430 (3)	C33—H38	0.9900
O13—C46	1.432 (3)	C34—H39	0.9900
O14—C38	1.428 (3)	C34—H40	0.9900
O14—C39	1.429 (3)	C35—H41	0.9900
O15—C40	1.422 (3)	C35—H42	0.9900
O15—C41	1.428 (3)	C36—H43	0.9900
O16—C43	1.432 (3)	C36—H44	0.9900
O16—C42	1.439 (3)	C37—H45	0.9900
O17—C44	1.423 (3)	C37—H46	0.9900
O17—C45	1.426 (3)	C38—H47	0.9900
N1—C2	1.147 (3)	C38—H48	0.9900
N2—C4	1.145 (3)	C39—H49	0.9900
N3—C6	1.152 (3)	C39—H50	0.9900
N4—C8	1.143 (3)	C40—H51	0.9900
N5—C10	1.147 (3)	C40—H52	0.9900
N6—C12	1.147 (3)	C41—H53	0.9900
N7—C14	1.150 (3)	C41—H54	0.9900
N8—C16	1.147 (3)	C42—H55	0.9900
C1—C3	1.360 (3)	C42—H56	0.9900
C1—C2	1.443 (3)	C43—H57	0.9900
C3—C4	1.433 (3)	C43—H58	0.9900
C5—C7	1.360 (3)	C44—H59	0.9900
C5—C6	1.436 (3)	C44—H60	0.9900
C7—C8	1.438 (3)	C45—H61	0.9900
C9—C11	1.364 (3)	C45—H62	0.9900
C9—C10	1.437 (3)	C46—H63	0.9900
C11—C12	1.441 (3)	C46—H64	0.9900
			
C4···C11^i^	3.371 (3)	N2···C12^i^	3.324 (3)
			
S1—Fe1—S3	87.51 (3)	O1—C17—H6	110.5
S1—Fe1—S4	151.95 (3)	C18—C17—H6	110.5
S3—Fe1—S4	90.01 (3)	H5—C17—H6	108.7
S1—Fe1—S2	90.01 (3)	O2—C18—H7	110.3
S3—Fe1—S2	170.42 (3)	C17—C18—H7	110.3
S4—Fe1—S2	87.84 (3)	O2—C18—H8	110.3
S1—Fe1—S2^i^	101.79 (2)	C17—C18—H8	110.3
S3—Fe1—S2^i^	94.92 (2)	H7—C18—H8	108.6
S4—Fe1—S2^i^	106.26 (2)	O2—C19—H9	110.3
S2—Fe1—S2^i^	94.64 (2)	C20—C19—H9	110.3
S5—Fe2—S8	84.05 (3)	O2—C19—H10	110.3
S5—Fe2—S7	154.50 (3)	C20—C19—H10	110.3
S8—Fe2—S7	90.07 (3)	H9—C19—H10	108.6
S5—Fe2—S6	89.71 (3)	O3—C20—H11	110.6
S8—Fe2—S6	165.16 (3)	C19—C20—H11	110.6
S7—Fe2—S6	89.83 (3)	O3—C20—H12	110.6
S5—Fe2—S8^ii^	106.02 (3)	C19—C20—H12	110.6
S8—Fe2—S8^ii^	100.53 (2)	H11—C20—H12	108.8
S7—Fe2—S8^ii^	99.45 (2)	O3—C21—H13	110.0
S6—Fe2—S8^ii^	94.12 (2)	C22—C21—H13	110.0
O7—Fe3—O6	175.32 (8)	O3—C21—H14	110.0
O7—Fe3—O3	85.97 (7)	C22—C21—H14	110.0
O6—Fe3—O3	90.30 (7)	H13—C21—H14	108.4
O7—Fe3—O2	95.17 (6)	O4—C22—H15	110.4
O6—Fe3—O2	86.44 (6)	C21—C22—H15	110.4
O3—Fe3—O2	73.20 (6)	O4—C22—H16	110.4
O7—Fe3—O5	96.00 (7)	C21—C22—H16	110.4
O6—Fe3—O5	85.49 (7)	H15—C22—H16	108.6
O3—Fe3—O5	145.53 (6)	O4—C23—H17	110.5
O2—Fe3—O5	140.31 (6)	C24—C23—H17	110.5
O7—Fe3—O4	88.91 (7)	O4—C23—H18	110.5
O6—Fe3—O4	87.24 (6)	C24—C23—H18	110.5
O3—Fe3—O4	71.75 (6)	H17—C23—H18	108.7
O2—Fe3—O4	144.31 (6)	O5—C24—H19	110.5
O5—Fe3—O4	73.88 (6)	C23—C24—H19	110.5
O7—Fe3—O1	81.72 (7)	O5—C24—H20	110.5
O6—Fe3—O1	102.96 (7)	C23—C24—H20	110.5
O3—Fe3—O1	142.47 (6)	H19—C24—H20	108.7
O2—Fe3—O1	72.80 (6)	O5—C25—H21	110.6
O5—Fe3—O1	71.34 (6)	C26—C25—H21	110.6
O4—Fe3—O1	142.68 (6)	O5—C25—H22	110.6
C1—S1—Fe1	103.91 (8)	C26—C25—H22	110.6
C3—S2—Fe1	104.00 (8)	H21—C25—H22	108.7
C3—S2—Fe1^i^	101.30 (7)	O1—C26—H23	110.6
Fe1—S2—Fe1^i^	85.36 (2)	C25—C26—H23	110.6
C5—S3—Fe1	103.81 (8)	O1—C26—H24	110.6
C7—S4—Fe1	103.51 (8)	C25—C26—H24	110.6
C9—S5—Fe2	103.76 (8)	H23—C26—H24	108.8
C11—S6—Fe2	103.57 (8)	O8—C27—H25	109.8
C13—S7—Fe2	103.41 (8)	C28—C27—H25	109.8
C15—S8—Fe2	104.36 (8)	O8—C27—H26	109.8
C15—S8—Fe2^ii^	101.71 (7)	C28—C27—H26	109.8
Fe2—S8—Fe2^ii^	79.47 (2)	H25—C27—H26	108.2
C26—O1—C17	114.36 (18)	O9—C28—H27	109.9
C26—O1—Fe3	115.32 (13)	C27—C28—H27	109.9
C17—O1—Fe3	112.03 (13)	O9—C28—H28	109.9
C18—O2—C19	113.03 (17)	C27—C28—H28	109.9
C18—O2—Fe3	114.53 (13)	H27—C28—H28	108.3
C19—O2—Fe3	112.99 (14)	O9—C29—H29	109.9
C20—O3—C21	119.59 (18)	C30—C29—H29	109.9
C20—O3—Fe3	116.11 (14)	O9—C29—H30	109.9
C21—O3—Fe3	119.02 (14)	C30—C29—H30	109.9
C22—O4—C23	112.99 (17)	H29—C29—H30	108.3
C22—O4—Fe3	111.40 (13)	O10—C30—H31	109.8
C23—O4—Fe3	111.10 (14)	C29—C30—H31	109.8
C25—O5—C24	113.00 (18)	O10—C30—H32	109.8
C25—O5—Fe3	113.80 (13)	C29—C30—H32	109.8
C24—O5—Fe3	112.14 (13)	H31—C30—H32	108.3
Fe3—O6—H2	124 (2)	O10—C31—H33	109.1
Fe3—O6—H1	120 (2)	C32—C31—H33	109.1
H2—O6—H1	113 (3)	O10—C31—H34	109.1
Fe3—O7—H4	121.0 (19)	C32—C31—H34	109.1
Fe3—O7—H3	128 (2)	H33—C31—H34	107.8
H4—O7—H3	111 (3)	O11—C32—H35	110.2
C27—O8—C36	113.86 (17)	C31—C32—H35	110.2
C28—O9—C29	112.48 (18)	O11—C32—H36	110.2
C31—O10—C30	112.41 (18)	C31—C32—H36	110.2
C32—O11—C33	113.93 (18)	H35—C32—H36	108.5
C34—O12—C35	112.55 (18)	O11—C33—H37	109.0
C37—O13—C46	113.73 (18)	C34—C33—H37	109.0
C38—O14—C39	113.71 (18)	O11—C33—H38	109.0
C40—O15—C41	111.87 (17)	C34—C33—H38	109.0
C43—O16—C42	114.19 (17)	H37—C33—H38	107.8
C44—O17—C45	112.39 (18)	O12—C34—H39	109.8
C3—C1—C2	120.5 (2)	C33—C34—H39	109.8
C3—C1—S1	122.47 (17)	O12—C34—H40	109.8
C2—C1—S1	117.02 (17)	C33—C34—H40	109.8
N1—C2—C1	177.9 (3)	H39—C34—H40	108.2
C1—C3—C4	122.3 (2)	O12—C35—H41	110.1
C1—C3—S2	119.58 (17)	C36—C35—H41	110.1
C4—C3—S2	118.11 (17)	O12—C35—H42	110.1
N2—C4—C3	179.0 (3)	C36—C35—H42	110.1
C7—C5—C6	120.8 (2)	H41—C35—H42	108.4
C7—C5—S3	120.88 (17)	O8—C36—H43	109.3
C6—C5—S3	118.28 (17)	C35—C36—H43	109.3
N3—C6—C5	179.2 (3)	O8—C36—H44	109.3
C5—C7—C8	121.3 (2)	C35—C36—H44	109.3
C5—C7—S4	121.04 (18)	H43—C36—H44	107.9
C8—C7—S4	117.69 (17)	O13—C37—H45	109.0
N4—C8—C7	179.0 (3)	C38—C37—H45	109.0
C11—C9—C10	122.2 (2)	O13—C37—H46	109.0
C11—C9—S5	121.24 (17)	C38—C37—H46	109.0
C10—C9—S5	116.52 (17)	H45—C37—H46	107.8
N5—C10—C9	177.5 (2)	O14—C38—H47	110.1
C9—C11—C12	120.4 (2)	C37—C38—H47	110.1
C9—C11—S6	120.49 (17)	O14—C38—H48	110.1
C12—C11—S6	119.10 (17)	C37—C38—H48	110.1
N6—C12—C11	178.1 (2)	H47—C38—H48	108.4
C15—C13—C14	119.2 (2)	O14—C39—H49	108.9
C15—C13—S7	122.57 (18)	C40—C39—H49	108.9
C14—C13—S7	118.24 (17)	O14—C39—H50	108.9
N7—C14—C13	178.3 (3)	C40—C39—H50	108.9
C13—C15—C16	122.6 (2)	H49—C39—H50	107.7
C13—C15—S8	119.59 (17)	O15—C40—H51	110.0
C16—C15—S8	117.83 (17)	C39—C40—H51	110.0
N8—C16—C15	178.1 (2)	O15—C40—H52	110.0
O1—C17—C18	106.28 (19)	C39—C40—H52	110.0
O2—C18—C17	106.92 (18)	H51—C40—H52	108.4
O2—C19—C20	106.86 (18)	O15—C41—H53	110.1
O3—C20—C19	105.52 (18)	C42—C41—H53	110.1
O3—C21—C22	108.28 (19)	O15—C41—H54	110.1
O4—C22—C21	106.68 (18)	C42—C41—H54	110.1
O4—C23—C24	106.14 (19)	H53—C41—H54	108.4
O5—C24—C23	106.27 (19)	O16—C42—H55	109.2
O5—C25—C26	105.92 (18)	C41—C42—H55	109.2
O1—C26—C25	105.49 (18)	O16—C42—H56	109.2
O8—C27—C28	109.37 (19)	C41—C42—H56	109.2
O9—C28—C27	108.80 (19)	H55—C42—H56	107.9
O9—C29—C30	108.8 (2)	O16—C43—H57	110.0
O10—C30—C29	109.27 (19)	C44—C43—H57	110.0
O10—C31—C32	112.7 (2)	O16—C43—H58	110.0
O11—C32—C31	107.37 (19)	C44—C43—H58	110.0
O11—C33—C34	112.8 (2)	H57—C43—H58	108.3
O12—C34—C33	109.38 (19)	O17—C44—H59	110.1
O12—C35—C36	108.10 (19)	C43—C44—H59	110.1
O8—C36—C35	111.76 (19)	O17—C44—H60	110.1
O13—C37—C38	113.0 (2)	C43—C44—H60	110.1
O14—C38—C37	108.11 (18)	H59—C44—H60	108.4
O14—C39—C40	113.3 (2)	O17—C45—H61	110.0
O15—C40—C39	108.38 (19)	C46—C45—H61	110.0
O15—C41—C42	108.18 (18)	O17—C45—H62	110.0
O16—C42—C41	112.00 (19)	C46—C45—H62	110.0
O16—C43—C44	108.68 (19)	H61—C45—H62	108.3
O17—C44—C43	107.9 (2)	O13—C46—H63	109.9
O17—C45—C46	108.6 (2)	C45—C46—H63	109.9
O13—C46—C45	108.75 (19)	O13—C46—H64	109.9
O1—C17—H5	110.5	C45—C46—H64	109.9
C18—C17—H5	110.5	H63—C46—H64	108.3
			
C2—C1—C3—C4	1.9 (3)	C36—O8—C27—C28	156.54 (19)
S1—C1—C3—C4	−177.16 (16)	C29—O9—C28—C27	174.85 (18)
C2—C1—C3—S2	179.80 (16)	O8—C27—C28—O9	−66.3 (2)
S1—C1—C3—S2	0.7 (3)	C28—O9—C29—C30	−170.43 (19)
C6—C5—C7—C8	1.2 (3)	C31—O10—C30—C29	−168.56 (19)
S3—C5—C7—C8	−178.97 (17)	O9—C29—C30—O10	71.8 (2)
C6—C5—C7—S4	−179.64 (16)	C30—O10—C31—C32	85.3 (2)
S3—C5—C7—S4	0.2 (3)	C33—O11—C32—C31	−173.8 (2)
C10—C9—C11—C12	−2.2 (3)	O10—C31—C32—O11	64.8 (3)
S5—C9—C11—C12	178.91 (16)	C32—O11—C33—C34	87.5 (2)
C10—C9—C11—S6	177.08 (16)	C35—O12—C34—C33	178.1 (2)
S5—C9—C11—S6	−1.8 (3)	O11—C33—C34—O12	65.9 (3)
C14—C13—C15—C16	−2.5 (3)	C34—O12—C35—C36	166.9 (2)
S7—C13—C15—C16	176.80 (16)	C27—O8—C36—C35	−80.4 (2)
C14—C13—C15—S8	179.67 (16)	O12—C35—C36—O8	−65.5 (3)
S7—C13—C15—S8	−1.0 (3)	C46—O13—C37—C38	−82.9 (2)
C26—O1—C17—C18	179.23 (18)	C39—O14—C38—C37	170.0 (2)
C19—O2—C18—C17	174.47 (18)	O13—C37—C38—O14	−68.0 (3)
O1—C17—C18—O2	−57.2 (2)	C38—O14—C39—C40	−87.2 (2)
C18—O2—C19—C20	−177.03 (18)	C41—O15—C40—C39	−178.36 (19)
C21—O3—C20—C19	165.03 (19)	O14—C39—C40—O15	−64.8 (3)
O2—C19—C20—O3	54.1 (2)	C40—O15—C41—C42	−165.94 (19)
C20—O3—C21—C22	133.0 (2)	C43—O16—C42—C41	82.2 (2)
C23—O4—C22—C21	−177.92 (19)	O15—C41—C42—O16	67.1 (2)
O3—C21—C22—O4	45.8 (2)	C42—O16—C43—C44	−156.26 (19)
C22—O4—C23—C24	174.18 (19)	C45—O17—C44—C43	−175.73 (19)
C25—O5—C24—C23	174.64 (19)	O16—C43—C44—O17	66.8 (2)
O4—C23—C24—O5	−60.5 (2)	C44—O17—C45—C46	166.12 (19)
C24—O5—C25—C26	−179.14 (18)	C37—O13—C46—C45	171.56 (19)
C17—O1—C26—C25	−172.13 (18)	O17—C45—C46—O13	−71.7 (2)
O5—C25—C26—O1	56.7 (2)		

Symmetry codes: (i) −*x*+1, −*y*+1, −*z*; (ii) −*x*, −*y*+1, −*z*.

### Hydrogen-bond geometry (Å, °)

**Table:**

*D*—H···*A*	*D*—H	H···*A*	*D*···*A*	*D*—H···*A*
O6—H1···O8	0.78 (3)	1.96 (3)	2.726 (3)	171 (3)
O6—H2···O10	0.76 (3)	2.13 (3)	2.882 (2)	173 (3)
O7—H3···O13	0.76 (3)	2.04 (3)	2.779 (2)	164 (3)
O7—H4···O16	0.81 (3)	1.94 (3)	2.740 (2)	170 (3)
